# Closed Incision Negative Pressure Therapy Achieves Better Outcome Than Standard Wound Care: Clinical Outcome and Cost-Effectiveness Analysis in Open Ventral Hernia Repair With Synthetic Mesh Positioning

**DOI:** 10.7759/cureus.8283

**Published:** 2020-05-26

**Authors:** Leo Licari, Sofia Campanella, Claudia Carolla, Simona Viola, Guseppe Salamone

**Affiliations:** 1 Surgical, Oncological and Oral Sciences, University of Palermo, Palermo, ITA; 2 Surgical, Oncological and Oral Sciences, Policlinico Universitario P. Giaccone, University of Palermo, Palermo, ITA; 3 Biological, Chemical and Pharmaceutical Sciences and Technologies, University of Palermo, Palermo, ITA

**Keywords:** closed incision negative pressure therapy, ventral hernia repair, outcome, cost-effectiveness, abdominal surgery, surgical site infections, surgical site occurrences, guidelines

## Abstract

Background

Surgical site infections (SSIs) and surgical site occurrences (SSOs) are frequent post-operative complications that are dependent on the presence of different risk factors. The use of closed incision negative pressure therapy (ciNPT) is considered a measure by the WHO guidelines for prevention of SSIs. The prevention of SSOs is an extremely important issue in the ventral hernia repair (VHR) surgical field. SSO onset not only affects the patient’s quality of life, but can also cause the onset of life-threatening conditions that may require re-hospitalization, re-intervention and often mesh removal. Such outcome can become extremely costly, contributing to increased health care costs for the patient as well as the hospital. This study aims (1) to describe the epidemiological characteristics of SSOs following VHR in our experience; (2) to compare the post-operative outcomes of those who underwent VHR with synthetic mesh when treated with standard wound care (SWC) using gauze dressings vs ciNPT, and finally (3) to perform a spending review of the ciNPT in the hypothesis of its application after VHR with use of synthetic prosthetic material; financial savings including cost-effectiveness were investigated in terms of prevention of wound complications.

Materials and methods

A retrospective review was performed on patients who underwent open VHR with synthetic mesh positioning by analyzing the hospital medical records between January 2015 and December 2017, with a primary focus on high risk post-operative complications, such as age > 65, pre-existed wound infection, pulmonary diseases, BMI > 25 kg/m^2^, malnutrition, ascites, hypertension, diabetes, active smoking, previous radiation therapy, steroid use, pharmacological immunosuppression, chronic inflammatory diseases. In the final analysis, the outcomes of 70 patients who received ciNPT and 110 who were managed with using adherent gauze dressings were compared.

Results

Nine (12.8%) patients in the ciNPT group and 48 (43.6%) in the control group developed a wound complication (p < 0.0001). The relative risk (RR) was 0.29 (0.15 - 0.56), suggesting that infection is less likely to occur in ciNPT-treated incisions, compared with standard wound care. The differences observed between the superficial infection rate and the deep infection rate were significant with p respectively 0.0006 and 0.04. Wound complications were reported in patients after discharge from the hospital. Fever was reported in 28.6% of patients in the ciNPT group vs 54.5% in the control group (p = 0.0006; RR (95% CI) 0.52 (0.35 - 0.79); OR (95% CI) 0.33 (0.18 - 0.63)); leukocytosis affected 21.4% of patients treated with ciNPT vs 45.4% of patients in the control group (p = 0.001; RR (95% CI) 0.47 (0.29 - 0.77); OR 0.33 (0.16 - 0.65)). ciNPT patients had shorter hospitalization stay than control group (3 ± 1.37 vs 6 ± 2.39; p < 0.00001). The cost-effectiveness deterministic analysis estimated that if the ciNPT is routinely adopted, the reduction of total costs would be €166’944.00 for 100 patients.

Conclusions

This study demonstrates that ciNPT use in high-risk populations following VHR with synthetic mesh positioning is associated with positive clinical and economic outcomes.

## Introduction

Postoperative complications in general surgical patients are common and can lead to life-threatening conditions.

Surgical site infections (SSIs) are defined by the Centers for Disease Control and Prevention (CDC) as an infection that occurs in the part of the body where the surgery took place and includes superficial, deep, and organ space SSIs [1]. Surgical site occurrences (SSOs) are defined by the Ventral Hernia Working Group (VHWG) as any SSI as well as wound cellulitis, non-healing incisional wound, fascial disruption, skin or soft tissue ischemia, skin or soft tissue necrosis, wound serous or purulent drainage, stitch abscess, seroma, hematoma, infected or exposed mesh, or development of an enterocutaneous fistula [2].

SSOs are observed in up to 60% of inpatient surgical procedures [3]; SSIs are the most relevant postoperative complications with a reported incidence ranging from 15 to 37% [4].

The risk factors related to SSOs are known and certain comorbidities are directly related to the increased risk, including obesity, malignancies, malnutrition, poor controlled diabetes, tobacco use, emergency surgery, immunodeficiency syndromes [4].

The management of SSOs is important when considering the economic and organizational burden that these have on the health care system.

As a reminder, it was demonstrated that the financial impact of the health care-related infections accounts for 33.7% of US health spending per year [5].

The magnitude that the issue has on our health care system as well as the patient is very significant and this is demonstrated by the continued interest in defining the risk factors and in planning the strategies for prevention. Prevention is focused upon three moments of the surgical operation that the World Health Organization (WHO) summarize in pre-, intra- and post-operative measures. Post-operative measures include the use of advanced techniques and materials to help prevent SSIs [6].

The use of negative pressure wound therapy on clean, closed incisions is a procedure that has demonstrated to be beneficial for surgical site complications, including wound dehiscence. Its use is recommended not only for prevention of SSIs but also for helping to hold the incision edges together, homogeneously distributing the laterally orient forces across the wound, reducing edema and stimulating granulation tissue for wound healing [7,8].

Although the literature is replete with data regarding the use of the closed-incision negative pressure therapy (ciNPT) in surgery, few authors have focused on its use following ventral hernia repair (VHR) with synthetic meshes. This surgical field is very delicate and requires extra attention with regard to wound care post-operatively. The onset of SSOs could be responsible for significant quality of life (QoL) alterations and most of all the onset of life-threatening complications that potentially require re-operations and explantation of the mesh.

This study aims (1) to describe the epidemiological characteristics of SSOs following VHR in our experience, (2) to compare the post-operative outcomes of those who underwent VHR with synthetic mesh when treated with standard wound care (SWC) using gauze dressings vs ciNPT, and finally (3) to perform a spending review of the ciNPT in the hypothesis of its application after VHR with use of synthetic prosthetic material; financial savings including cost-effectiveness were investigated in terms of prevention of wound complications.

## Materials and methods

**Figure 1:**
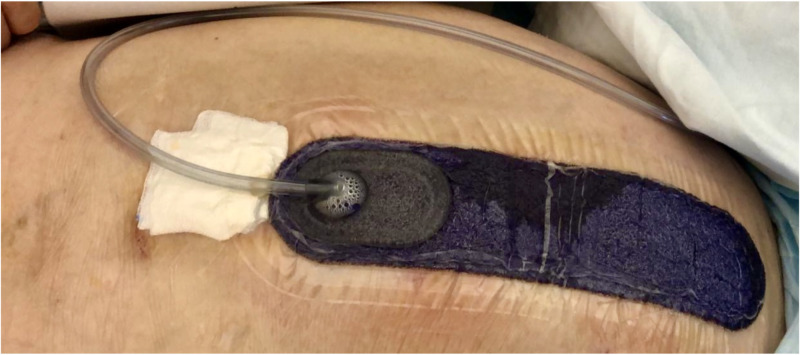
PrevenaTM Peel & PlaceTM Dressing, KCI. The photo shows the Prevena dressing at the time of removal. The white gauze is positioned to show the transparency of the drain tube. (Source: photo from personal archive)

Patients admitted to University Hospital of Palermo who underwent elective open VHR with synthetic mesh positioning between January 2015 and December 2017 were retrospectively identified in a database and the data collected were retrospectively reviewed. Patients’ medical and surgical records were collected from the charts and surgical registries. Approval by the Regional Ethics Review Board was obtained (ID number 0-201-9-05). Inclusion criteria for patients’ selection were the presence of one or more of the following factors: age > 65, pre-existed wound infection, pulmonary disease, BMI > 25 kg/m^2^, malnutrition, ascites, hypertension, diabetes, active smoking, previous radiation therapy, steroid use, pharmacological immunosuppression, chronic inflammatory diseases. All operations were performed by skilled general surgeons with patients under general anesthesia, receiving preoperative antibiotic prophylaxis ceftazidime 2 g at least 1 hr before the skin incision; if a patient had a β lactam allergy, clindamycin 900 mg IV and gentamicin 4 mg/kg IV based on actual body weight were administered. The WHO preoperative checklist was routinely performed; preoperative prevention skills were obtained according to the WHO and CDC recommendations. The closed-incision negative pressure therapy system (ciNPT) (Prevena^TM^ Peel & Place^TM^ Dressing, KCI, San Antonio, TX) was applied in the operating room, following skin closure, under sterile conditions (Figure 1).

The system permitted the setting of continuous negative pressure at -125 mmHg. The system was kept in place for seven days after its positioning. The dressing was then removed after seven days, often removed during the first follow-up control visit. After the removal, description of the wound and any complications were noted. All patients were followed for a period of 90 days after the operation; patients were visited weekly in the first month and then monthly for two months. All patients were compliant on follow-up. The complication rate of patients treated with ciNPT was compared with that of the standard of care dressing. The standard of care dressing consisted of dry or moistened gauze, abdominal pads, adhesive dressings, or skin adhesives, applied in the operating room, immediately after the skin closure and changed every 48 hours during routine in-hospital visits.

Regarding cost-effective analysis, a deterministic analysis was performed on the basis of real data from our population and according to financial data provided by the National Health System dataset. Costs are reported in euros (€).

Data were analyzed in Excel 2016 and IBM SPSS software, version 21 (IBM Corp., Armonk, NY, USA). Quantitative variables were collected as count and percentage. The mean and median were obtained for continuous variables. Comparisons of continuous variables were made using Student’s t test for independent means or the Mann-Whitney test where appropriate. A comparison of categorical variables was made with the Chi-squared (χ2) test or Fisher’s exact test. Relative Risk (RR) 95% CI and Odds Ratio (OR) 95% CI were calculated according to Altman. The statistical significance level was set to a p-value < 0.05.

## Results

Between January 2015 and December 2017, a total of 274 patients were admitted to the Policlinico “Paolo Giaccone” Hospital and underwent a surgical operation for ventral hernia repair. Ninety-four patients were excluded because they were repaired laparoscopically or without use of synthetic mesh and/or lacked significant risk factors for development of SSOs. One-hundred-eighty patients were selected for the study. Of these, 70 patients were treated with ciNPT, whereas 110 were treated with SWC. The surgical operation performed was elective open ventral hernia repair with synthetic mesh positioning. The techniques used were the IPOM in 107 patients and the Rives technique in 73 patients. The wounds were classified as “clean” in all the patients, according to the CDC wounds classification [9].

There were no significant differences in demographic data between the two groups (Table 1).

**Table 1:** Demographic data ASA: American Society of Anesthesiologists; DM: Diabetes Mellitus; ciNPT: closed-incision Negative Pressure Therapy; SWC: Standard Wound Care; ns: not significant; SD: Standard Deviation.

	ciNPT	SWC	p
n (%)	70 (38.8%)	110 (61.2%)	
Age (mean; SD)	73.3 (± 12.67)	75 (± 13.52)	n.s.
Sex			
M	63%	64%	n.s.
F	37%	36%	n.s.
BMI (kg/m^2^) (median; SD)	28 (± 2)	26 (±5)	n.s.
Smokers	59%	62%	n.s.
Comorbidities			
Hypertension (%)	67	70	n.s.
DM Type 2 (%)	49	52	n.s.
Cardiovascular disease (%)	33	35	n.s.
Controlled liver disease (%)	12	6	n.s.
Pulmonary disease (%)	25	21	n.s.
Inflammatory bowel disease (%)	10	15	n.s.
Pre-existed wound infection (%)	6	10	n.s.
Previous radiation therapy (%)	7	20	n.s.
ASA score (%)			
II	25	27	n.s.
III	71	63	n.s.
IV	4	10	n.s.
Intraoperative data			
Operation time (min) (mean; SD)	90.2 (±45)	88.3 (±37)	n.s.
Hernia size (cm^2^) (mean; SD)	138.8 (±2.6)	142.3 (±3.5)	n.s.
Width of the defect (mean; SD)	10 (±5)	10 (±5)	n.s.

The overall complication rate was 31.6% (57 patients). Nine patients (12.8%) belonged to the ciNPT group and 48 patients (43.6%) belonged to the SWC group (p < 0.0001). The RR (95% CI) was 0.29 (0.15 - 0.56) and the OR (95% CI) was 0.19 (0.09 - 0.42). The differences observed between the superficial infection rate and the deep infection rate were significant with p value of 0.0006 and 0.04, respectively (Table 2). All the major complications occurred after patients' discharge.

**Table 2:** Complication rate ^§^ All complications occurred after discharge. ^†^ Minor complications occurred both during in-hospital stay - and they were responsible for prolonged hospitalization - and during follow-up time simultaneously to major complications. ciNPT: closed-incision Negative Pressure Therapy; SWC: Standard wound care; RR: Relative risk; OR: Odds ratio; CI: Confidence interval; WBC: White blood cells.

	ciNPT	SWC	p-value	RR (95% CI)	OR (95% CI)
Overall major complications^§^ n (%)	9 (12.8%)	48 (43.6%)	<0.00001	0.29 (0.15 - 0.56)	0.19 (0.09 - 0.42)
Seroma	4 (5.7%)	12 (10.9%)	0.23	0.52 (0.18 - 1.56)	0.49 (0.15 - 1.60)
Superficial infection	3 (4.3%)	25 (22.7%)	0.0006	0.19 (0.06 - 0.60)	0.15 (0.04 - 0.53)
Deep infection	0	7 (6.4%)	0.04	0.10 (0.01 - 1.79)	0.10 (0.01 - 1.74)
Wound dehiscence	2 (2.9%)	4 (3.6%)	0.7	0.78 (0.15 - 4.17)	0.78 (0.14 - 4.37)
Minor complication rate^†^					
Fever > 37.5°C	20 (28.6%)	60 (54.5%)	0.0006	0.52 (0.35 - 0.79)	0.33 (0.18 - 0.63)
Leukocytosis WBC > 11 x 10^3^/uL	15 (21.4%)	50 (45.4%)	0.001	0.47 (0.29 - 0.77)	0.33 (0.16 - 0.65)

Table 2 outlines differences in minor complication rates among the two groups; these were statistically significant. In detail, fever complicated the normal course of the patients in the ciNPT group in 28.6% of cases vs 54.5% of control group (p = 0.0006; RR (95% CI) 0.52 (0.35 - 0.79); OR (95% CI) 0.33 (0.18 - 0.63)); leukocytosis affected 21.4% of patients treated with ciNPT vs 45.4% of patients belonged to the control group (p = 0.001; RR (95% CI) 0.47 (0.29 - 0.77); OR 0.33 (0.16 - 0.65)). Minor complications occurred both during in-hospital stay - and they were responsible of prolonged hospitalization - and during follow-up time simultaneously to major complications.

Table 3 reports the mean cost of treatment per patient, including the cost of incisional hernia repair, the cost of the mesh, the cost of inpatient management, the cost of the Prevena^TM^ ciNPT and the cost of the standard of care dressing per patient. Mean in-hospital stay was shorter in the ciNPT group compared to control group (3 ± 1.37 vs 6 ± 2.39; p < 0.00001). The total estimated cost per patient in each group is €4,230.00 for ciNPT patients and €5,695.00 for SWC patients, respectively.

**Table 3:** Items cost analysis (costs are in euros € per patient) ciNPT: closed-incision Negative Pressure Therapy; SWC: Standard wound care; SD: Standard deviation.

	ciNPT	SWC	p value
Incisional hernia intervention	1048	1048	
In hospital stay cost per day	600	600	
Mesh-related cost (mean)	1007	1007	
Prevena^TM^	375	0	
Standard of care dressing	0	40	
Mean in-hospital stay (days) (mean; SD)	3 ± 1.37	6 ± 2.39	<0.00001
Total (mean; SD)	4230 ± 1928.56	5695 ± 3142.27	0.02

Table 4 demonstrates the strategies used for complications management. Total re-hospitalization rate was 7.2% (13 patients). Two patients belonged to the ciNPT group (2.8%) and 11 patients belonged to the SWC group (10%). The mean in-hospital stay after re-admission was similar for both groups 5 ± 2 days. The treatment choice was significantly different in terms of re-operation rate with surgical toilette: no patients were treated in ciNPT group vs seven patients treated in SWC group with p = 0.04, RR (95% CI) 0.10 (0.01-1.80) and OR (95% CI) 0.26 (0.06-1.23). Four of seven patients in SWC group required negative pressure wound therapy (NPWT) following surgical toilette. Moreover, NPWT was used in two patients of the ciNPT group. Their wounds presented with superficial cutis dehiscence no longer than 5 cm in length and no deeper than 5 cm; surgical toilette in operating room was not required. The bedside open NPWT was considered suitable. Management of NPWT required a mean changing time of the therapy of 48-72 hours for at least five changes in both groups.

**Table 4:** Cost analysis of complications after discharge (costs are in euros €) ciNPT: closed-incision Negative Pressure Therapy; SWC: Standard wound care; RR: Relative risk; OR: Odds ratio; CI: Confidence interval; NPWT: Negative pressure wound therapy; pt: patient.

	ciNPT	SWC	p value	RR (95% CI)	OR (95% CI)	Costs (€)
In-patient management						
Re-hospitalization rate	2 (2.8%)	11 (10%)	0.08	0.28 (0.06-1.25)	0.26 (0.06-1.23)	
Mean in-hospital stay (day)	5 ± 1.7	5 ± 2.2	0.95	0.98 (0.35-5.21)	0.97 (0.32-5.20)	600 per day
Treatment						
Re-operation with surgical toilette	0	7 (6.4%)	0.04	0.10 (0.01-1.80)	0.26 (0.06-1.23)	200 per pt
Amount per pt (€)	-	3200				
NPWT (5 dressings)	2 (1.8%)	4 (3.6%)	0.77	0.78 (0.15-4.18)	0.77 (0.14-4.37)	775 per pt
Amount per pt (€)	3775	3775				
Total amount (€)	7550	37500				
Out-patient management						
Outpatient management rate	7 (10%)	37 (33.6%)	0.0003	0.3 (0.14-0.63)	0.23 (0.09-0.52)	
Treatment						
Wound dressings	7 (10%)	37 (33.6%)	0.0003	0.3 (0.14-0.63)	0.23 (0.09-0.52)	40 per pt
Amount per pt (€)	40	40				
Total amount (€)	280	1480				
Overall amount (€)	7830	38980				

Overall, outpatient management rate of post-operative complications was 24.4% (44 patients). Seven patients belonged to the ciNPT group (10%) and 37 patients belonged to SWC group (33.6%). A significant difference was found, with p = 0.0003, RR (95% CI) 0.3 (0.14-0.63) and OR (95% CI) 0.23 (0.09-0.52). Patients were treated with standard wound care using gauze dressings in both groups.

In-hospital stay data including the cost per day if complications occurred and the costs of the complications management were collected and detailed in Table 4. The overall estimated cost of complication management is €2,610.00 per year in ciNPT group and €12,993.30 per year in SWC group.

**Figure 2:**
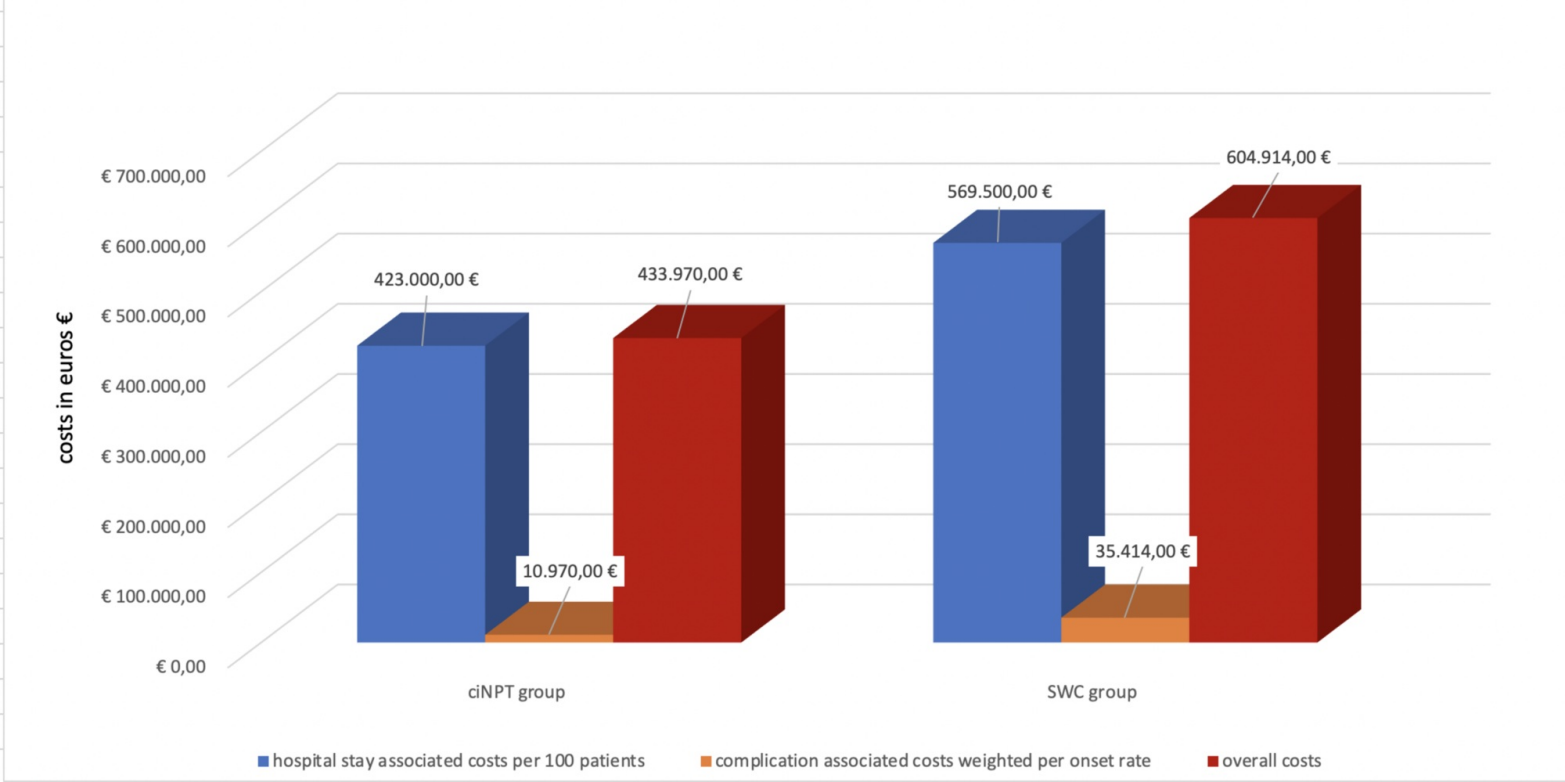
Cost-effective deterministic analysis for 100 patients undergoing open VHR with post-operative ciNPT treatment and with standard wound care. ciNPT: closed-incision Negative Pressure Therapy; SWC: Standard wound care; VHR: Ventral hernia repair. in blue: hospital stay-associated costs per 100 patients; in orange: complication-associated costs weighted per onset rate; in red: overall costs.

The cost-effectiveness deterministic analysis showed a significant reduction of hospital costs in a setting of 100 people treated with ciNPT vs standard wound care. In particular, when considering 100 patients, hospital stay associated cost falls between €569,500.00 (SWC) to €423,000.00 (ciNPT) (saving €146,500.00) while complication-associated cost weighted per onset rate falls between €35,414.00 (SWC) to 10,970.00 (ciNPT) (saving €24,444.00). Overall, if the ciNPT was routinely adopted, the reduction of the total costs is estimated to be €170,944.00 for 100 patients (Figure 2).

## Discussion

Based on our analysis, the overall rate for postoperative wound complications in patients treated with ciNPT system is significantly lower than patients treated with SWC. Our results demonstrate that the superficial infection rate and the deep infection rate are significantly lower in the ciNPT group compared to the standard of care group. The relative risk for complications onset decreased by 29% with ciNPT use (RR 0.29 95% CI 0.15-0.56). Our data further supports the use of ciNPT to prevent wound complications as previously reported in literature.

In fact, a recent meta-analysis by Tran et al. showed the efficacy of ciNPT in abdominal wall reconstruction. In a total of 1723 patients (681 patients treated with ciNPT and 1042 patients in control group), they demonstrated that the risk of surgical site infection and of wound dehiscence decreases by 51% with ciNPT use respectively RR 0.51 (95% CI 0.41-0.63) and RR 0.51 (95% CI 0.34-0.76) [10].

In the study by Tran et al., the overall rate of surgical site infection and of wound dehiscence was respectively 15% in ciNPT group vs 28% in control group and 8% in ciNPT group vs 15% in control group; the p-value was < 0.001 in both the cases.

The ciNPT system is applied in the operative room under sterile conditions, and remains in place without manipulation for a total of seven days post-operatively. The decreased frequency in dressing changes and the use of negative pressure therapy may relate to lower SSI rate compared to control group. Also, the shorter hospitalization time in ciNPT group was related to the decreased incidence of minor complications post-operatively.

The cost-effective analysis of ciNPT use in high-risk VHR patients demonstrated that the estimated overall cost of ciNPT use is lower than the control group because ciNPT patients have shorter hospitalization time. In this way, the estimated cost is €4,230.00 in ciNPT group vs €5,695.00 in control group. The complications management costs were estimated to be €2,610.00 per year in ciNPT group and €12,993.30 per year in control group. This potentially means cost savings of €10,383.30 per year if ciNPT would be used routinely in high-risk VHR patients. The cost-effectiveness analysis showed that in a sample of 100 patients, the total cost saving would be €170,944.00 if ciNPT would be used routinely in high-risk populations.

In a paper, Chopra et al., in 2015, stated that, “The clinical relevance of an innovation is no longer enough to justify acquisition costs” [11]. They demonstrated that the use of ciNPT use in a high-risk population is a beneficial cost-saving measure. The report stated that in their 829 patients undergoing abdominal wall reconstruction, ciNPT use resulted in an estimated cost savings of $1,542.52 and could be a cost-effective option when the estimated SSI rate is above 16% for the patient population.

In our opinion, further studies are necessary to analyze the weighted risk of multiple risk factors on the cumulative risk for post-operative complications in order to identify an easy-to-use and detailed scoring system to better stratify the patients; this could be useful to address the ciNPT use towards selected patients.

This study has several limitations, first of all because it is a retrospective analysis with the bias that it should carry on; second because of the small numbers of the patients in the study.

## Conclusions

The study shows the efficacy and feasibility of ciNPT use in high-risk population following open VHR with mesh positioning. The analysis demonstrates that ciNPT significantly decreases post-operative morbidities such as superficial and deep infections in high-risk patients following open VHR with mesh positioning and that the cost-effective analysis should justify its routine use in high-risk populations. Randomized controlled trials are needed to definitively validate the efficacy and feasibility of ciNPT use in patients undergoing open VHR with mesh positioning.
